# Expressions of Insulin-like Growth Factor System among Different Breeds Impact Piglets’ Growth during Weaning

**DOI:** 10.3390/ani13193011

**Published:** 2023-09-25

**Authors:** Mengying Dou, Md. Abul Kalam Azad, Yating Cheng, Sujuan Ding, Yang Liu, Bo Song, Xiangfeng Kong

**Affiliations:** 1 Key Laboratory of Agro-Ecological Processes in Subtropical Region, Hunan Provincial Key Laboratory of Animal Nutritional Physiology and Metabolic Process, National Engineering Laboratory for Pollution Control and Waste Utilization in Livestock and Poultry Production, Institute of Subtropical Agriculture, Chinese Academy of Sciences, Changsha 410125, China; 18437958239@163.com (M.D.); azad.fpe@gmail.com (M.A.K.A.); chengyating20@mails.ucas.edu.cn (Y.C.); jiayousujuan@isa.ac.cn (S.D.); liuyang@stu.njau.edu.cn (Y.L.); songbo20@mails.ucas.ac.cn (B.S.); 2 College of Advanced Agricultural Sciences, University of Chinese Academy of Sciences, Beijing 100008, China

**Keywords:** Chinese indigenous pig, insulin-like growth factor, receptor, binding protein, weaning

## Abstract

**Simple Summary:**

Animal growth depends on various factors, such as breed, nutrition, growth hormones, physiological stage, and environment. Therefore, the decrease in piglets’ growth after weaning is not only related to a decrease in feed intake but may also be related to the breed and growth hormones. Thus, the present study investigated the insulin-like growth factor (IGF) system components in Chinese indigenous pig breeds (Taoyuan black and Xiangcun black pigs) and commercial pig breeds (Duroc pigs) during weaning. Our findings indicated that weaning can affect the expressions of IGF system components in piglets, and the expressions of IGF system components in response to weaning stress differed in the three pig breeds. The differences in IGF system components in the three pig breeds might be related to their anti-stress tolerance characteristics.

**Abstract:**

The present study investigated the insulin-like growth factors (IGFs) and their receptors and binding proteins among three pig breeds during weaning. Sixty Duroc (DR), Taoyuan black (TYB), and Xiangcun black (XCB) piglets (20 piglets per breed) were selected at 21 and 24 (3 days of post-weaning) days of age to analyze organ indices, plasma concentrations of IGF and IGF-binding proteins (IGFBPs) using ELISA kits, and gene expression of IGF-system-related components in different tissues. The plasma IGFBP-3 concentration in TYB piglets was higher (*p* > 0.05) than in the XCB and DR piglets at 21 days of age. At 21 days of age, compared with the DR piglets, the *IGF-1* expression was lower (*p* < 0.05) in the kidney, but it was higher (*p* < 0.05) in the spleen of XCB and TYB piglets. At 24 days of age, the *IGF-1* expression was higher (*p* < 0.05) in the kidney of TYB piglets than in the XCB and DR piglets, while *IGFBP-3* in the stomach and *IGFBP-4* in the liver of XCB and TYB piglets were lower (*p* < 0.05) compared with the DR piglets. Weaning down-regulated (*p* < 0.05) *IGF-1* expression in the jejunum, spleen, and liver of piglets, while it up-regulated (*p* < 0.05) *IGFBP-3* expression in the stomach, *IGFBP-4* in the liver, *IGFBP-5* in the ileum, and *IGFBP-6* in the jejunum of DR piglets. Spearman’s correlation analysis showed a negative correlation (*p* < 0.05) between plasma IGFBP-2 and IGFBP-5 concentration and the organ indices of piglets. Collectively, there were significant differences in the IGF system components among the three pig breeds. The IGF system components were altered during weaning, which might be involved in weaning stress to decrease the growth of piglets.

## 1. Introduction

Growth rate is one of the most important traits in domestic animal breeding programs and is also considered an important factor in determining meat production [1]. Pork is a good and comparatively economical source of animal protein and is the most consumed meat in China [2]. Over the past decades, antibiotics have been used as growth promoters to prevent diseases and improve pork production in the pig industry [3]. Excessive use of antibiotics leads to increased antibiotic resistance in pathogenic bacteria and food residues in the pig industry [4]. In response, many European countries and China have banned antibiotics for animal growth promotion [5,6]. Therefore, understanding the growth and development profiles of pigs is of great significance to the improvement of pork production and pig breeding and developing alternatives to antibiotic growth promoters.

The growth and development of animals are regulated by numerous physiological networks [7]. Hormones are primarily responsible for the integrated communication of several physiological systems associated with growth and development regulation. Among these hormones, insulin-like growth factors (IGFs) are considered some of the key hormones of the growth axis [8]. IGFs are regulated by the IGF-signaling system, which includes IGFs, IGF receptors (IGF-Rs), and IGF-binding proteins (IGFBPs). The IGF system involves pleiotropic actions, such as mammalian growth, development, and metabolism [9]. IGFs are mainly produced by the liver and then released into the bloodstream, where they bind to IGFBPs and are transported to the target organs. IGFs bind to specific IGF-Rs located on the cell surface to activate downstream signaling pathways to promote cell proliferation and differentiation [10]. In particular, the IGF system is vital in initiating the connection between nutrition and the postnatal growth and development of various tissues and organs through secretory, paracrine, and endocrine actions [11,12]. Numerous environmental factors, such as nutritional status, stress, and temperature, significantly affect the IGF system [13]. In the commercial swine industry, suckling to weaning is the most stressful period for piglets due to the rapid changes in diets, physiology, and social environments, leading to weaning stress [14]. Research evidence indicated that weaning stress can limit the growth rate of piglets [15] and decrease serum IGF-1 and IGF-2 concentrations [16]. Hence, the changes in serum IGFs concentrations may affect the growth rate of weaned piglets. Previously, it has been found that piglets exhibited stress damage on the 3rd day after weaning [17]. However, the changes in the IGF system of piglets during this weaning period (3rd day after weaning) are still unclear.

It is well known that the Duroc (DR) pig, a commercial pig breed, has the characteristics of faster growth, higher lean meat ratio, and lower fat content [18]. The Chinese indigenous pig breeds are distinguished by presenting high prolificacy, better meat quality, and roughage feeding tolerance; however, the growth rate of these breeds is slower than that of the DR pigs [19]. Different pig breeds have been crossed to obtain heterosis and complementarity [20]. The Xiangcun black (XCB) pig is derived from a crossbreed of the DR pig as the paternal lines with the Taoyuan black (TYB) pig as the maternal lines. The XCB pig has the characteristics of strong adaptability to roughage feeding, higher intramuscular fat content, and a faster growth rate [21]. The availability of this pig breed provides a model for exploring the effects of the IGF system on the growth of pigs. Therefore, we hypothesized that the differences in growth and development of these three pig breeds might be related to the regulation of IGFs, IGF-Rs, and IGFBPs. Gene expression profiling studies have focused more attention on such factors as breed, age, and tissue specificity [11]. Previously, we found that weaning could decrease the body weight (BW) of DR, TYB, and XCB piglets [22]. Therefore, the present study focused on weaned DR, TYB, and XCB piglets to determine the changes in plasma IGF component concentrations and the expression levels of *IGFs*, *IGF-Rs*, and *IGFBPs* in different tissues of piglets. Additionally, weaning stress in piglets is mitigated by considering the management measures and nutritional interventions during pre- and post-weaning periods, such as improving the feeding conditions and the health of gestating sows in order to meet adequate colostrum and milk during lactation. Dietary interventions through feed additives with the functions to improve intestinal function and microbiota and enhance immunity (e.g., amino acids, probiotics, prebiotics), the morphology and palatability of diets, and the appropriate environment of the barn have also been used to prevent the weaning stress of piglets at the early age [23,24]. Thus, the present study will offer an informative perspective on the growth and development of different pig breeds and avenues to alleviate the adverse effects of weaning stress on the growth and development of piglets.

## 2. Materials and Methods

### 2.1. Animals and Experimental Design

Thirty litters of newborn DR, TYB, and XCB piglets (10 litters from each breed) were obtained from the sows with similar parity (2−3) and litter size (9−11). Two piglets close to the average BW of litter were chosen from each litter, and each pig breed consisted of 20 piglets (TYB and XCB piglets were half females and half castrated males, and DR piglets were all castrated males). The piglets only received sows’ milk during lactation. After weaning at 21 days of age, the piglets were fed four times (8:00, 12:00, 14:00, and 17:00) daily with the creep feed. The piglets had *ad libitum* access to feed and water during the trial. Piglets were kept in individual pens at a controlled temperature (23−26 °C) and forced-air ventilation. The piglets were not vaccinated with any vaccine during the trial.

### 2.2. Sample Collection

At 21 and 24 days of age, 10 piglets from each breed were weighed and slaughtered for sampling 12 h after the last feeding. The average BWs of DR, TYB, and XCB piglets were 6.01 ± 1.91, 5.06 ± 1.12, and 3.42 ± 1.12 kg at 21 days of age and 5.74 ± 1.59, 4.37 ± 1.78, and 3.22 ± 0.99 kg at 24 days of age, respectively. In order to obtain organ index and IGF system expressions in organs, piglets were exsanguinated after intravenous injection of 4% sodium pentobarbital (40 mg/kg). Blood (10 mL) samples were taken from the anterior vena cava into heparin sodium tubes (Aosaite, Heze, China) and centrifuged at 3500× *g* for 15 min at 4 °C to obtain plasma for IGF and IGFBP concentration analysis. The kidney, spleen, liver, *longissimus dorsi* (LD) muscle, stomach, duodenum, jejunum, and ileum were separated and weighed to calculate organ indices: organ weight (g)/body weight (kg). A 5-cm section of posterior segments of the duodenum, jejunum, and ileum was taken, placed in liquid nitrogen, and kept at –80 °C for total RNA extraction.

### 2.3. Measurement of IGF System Components in Plasma

The plasma concentrations of IGF-1 (HY-01223P1), IGF-2 (HY-01222P1), IGFBP-1 (HY-10336P1), IGFBP-2 (HY-10343P1), IGFBP-3 (HY-01221P1), and IGFBP-5 (HY-10331P1) were determined by the porcine ELISA kits (Shanghai Huyu, Shanghai, China). The absorbance values were read on the Multiscan Specturum Spectrophotometer (Tecan, Infinite M200 Pro, Männedorf, Switzerland).

### 2.4. Expression Analysis of IGF System Component Genes

The total RNA was extracted from the stomach, duodenum, jejunum, ileum, liver, kidney, spleen, and LD muscle tissues using the TRIzol reagent (AG21101; Accurate Biotechnology Co., Ltd., Changsha, China). The concentration and purity of the extracted RNA were determined using a NanoDrop 2000 spectrophotometer (Thermo Fischer Scientific, Waltham, MA, USA). The RNA was converted into cDNA by reverse transcription with PrimeScript RT Reagent Kit and gDNA Eraser (AG11705; Accurate Biotechnology Co., Ltd., Changsha, China). Real-time PCR assays were performed on the Light Cycler R 480 II Real-Time PCR System (Roche, Basel, Switzerland). The PCR conditions were as follows: 95 °C for 5 min, then 40 cycles of denaturation at 95 °C for 5 s and annealing at 60 °C for 30 s, and a final extension at 72 °C for 30 s. Primers for porcine IGF-system-related genes and housekeeping gene β-actin were synthesized by Tsingke Biotechnology Co., Ltd. (Beijing, China), and are shown in Table 1. Gene expression was normalized against β-actin and calculated using the 2^−ΔΔCt^ method [25].

### 2.5. Statistical Analysis

The data were analyzed by ANOVA using a 2 × 2 factorial treatment arrangement with SPSS 26.0 (SPSS Inc., Chicago, IL, USA). Breed and weaning were the two variables for this study, while dietary and environmental factors were consistent; thus, the model included weaning, breed, and their interactions. Multiple comparisons of means among different groups were performed with Tukey’s *post-hoc* test for valid interaction with a significance value at *p* < 0.05. Data are expressed as mean ± standard error of the mean (SEM). Spearman’s correlation analysis was performed to determine the relationships among plasma IGF concentrations, BW, and organ indices, as well as IGF system component gene expressions and organ indices in the three pig breeds.

## 3. Results

### 3.1. Weaning Effects on Organ Indices in Different Breeds of Piglets

Organ indices of the three breeds of piglets are listed in Table 2. The stomach and duodenum indices of the XCB piglets were higher (*p* < 0.05) compared with the TYB and DR piglets, regardless of weaning. Weaning increased (*p* < 0.05) the stomach index of piglets, regardless of pig breed. There was no interaction (*p* > 0.05) between the weaning and breed on the stomach, duodenum, jejunum, ileum, kidney, spleen, and liver indices.

### 3.2. Weaning Effects on Plasma IGF and IGFBP Concentration in Different Breeds of Piglets

Plasma IGF and IGFBP concentrations in the three breeds of piglets are presented in Table 3. The plasma IGF-1 concentration was higher (*p* < 0.05) in the XCB piglets compared with the TYB and DR piglets, regardless of weaning. At 21 days of age, plasma IGFBP-3 concentration was elevated (*p* < 0.05) in the TYB piglets compared with the XCB and DR piglets. Weaning reduced (*p* < 0.05) the plasma IGFBP-3 concentration in the TYB piglets. There was an interaction (*p* < 0.05) between weaning and breed on plasma IGFBP-3 concentration.

### 3.3. Weaning Effects on IGF-Related Gene Expressions in Different Breeds of Piglets

Gene expressions related to IGFs in the stomach, duodenum, jejunum, ileum, kidney, liver, spleen, and LD muscle of the three breeds of piglets are listed in Table 4. At 21 days of age, the *IGF-1* expression was down-regulated in the kidney and up-regulated in the spleen of XCB and TYB piglets compared with the DR piglets (*p* < 0.05). The *IGF-1* expression was down-regulated (*p* < 0.05) in the liver of XCB piglets compared with the TYB and DR piglets. At 24 days of age, the *IGF-1* expression was up-regulated (*p* < 0.05) in the kidney of TYB piglets compared with the XCB and DR piglets. At 21 days of age, the *IGF-1* expression was down-regulated (*p* < 0.05) in the jejunum, spleen, and liver of piglets, regardless of pig breed, as well as *IGF-2* expression in the stomach, kidney, and spleen of piglets. The *IGF-1* expression in the jejunum, kidney, spleen, and liver showed weaning–breed interactions (*p* < 0.05). However, there was no interaction (*p* > 0.05) between weaning and breed on *IGF-2* expression.

### 3.4. Weaning Effects on IGF Receptor (IGF-R) Gene Expressions in Different Breeds of Piglets

The expressions of *IGF-R* genes in the three breeds of piglets are presented in Table 5. The *IGF-1R* expression was up-regulated (*p* < 0.05) in the liver of XCB and TYB piglets compared with the DR piglets at 21 days of age, as well as in the XCB piglets compared with the TYB and DR piglets at 24 days of age. The *IGF-1R* expression was up-regulated (*p* < 0.05) in the ileum of XCB piglets compared with the TYB and DR piglets regardless of weaning, whereas it was down-regulated (*p* < 0.05) in the spleen (three breeds of piglets) and liver (TYB piglets) at 21 days of age compared with the 24 days of age. There was an interaction (*p* < 0.05) between weaning and breed on *IGF-1R* expression in the liver.

In comparison to the TYB and DR piglets, XCB piglets exhibited down-regulation (*p* < 0.05) of the *IGF-2R* expression in the kidney at 21 days of age; however, *IGF-2R* expression in the spleen and liver of XCB piglets was up-regulated (*p* < 0.05) regardless of weaning. The *IGF-2R* expression was up-regulated (*p* < 0.05) in the stomach and kidney of the three breeds of piglets. There was an interaction (*p* < 0.05) between weaning and breed on *IGF-2R* expression in the ileum and liver.

### 3.5. Weaning Effects on IGF-Binding Protein (IGFBP) Expression in Different Breeds of Piglets

The *IGFBP* expressions in the three breeds of piglets are shown in Table 6. The *IGFBP-1* expression in the liver was up-regulated, whereas it was very low in other tissues in the three breeds of piglets. Therefore, only the data for *IGFBP-1* expression in the liver are presented in Table 6. The *IGFBP-1* expression in the liver of XCB and TYB piglets was up-regulated (*p* < 0.05) compared with the DR piglets regardless of weaning. There was no interaction (*p* > 0.05) between weaning and breed for *IGFBP-1* expression in all tested tissues.

At 21 days of age, the *IGFBP-2* expression was up-regulated (*p* < 0.05) in the duodenum, ileum, kidney, and spleen of TYB piglets compared with the XCB and DR piglets. The *IGFBP-2* expression was up-regulated (*p* < 0.05) in the liver of XCB and TYB piglets compared with the DR piglets. At 24 days of age, the *IGFBP-2* expression was down-regulated (*p* < 0.05) in the LD muscle of XCB piglets compared with the DR piglets. Weaning down-regulated the *IGFBP-2* expression in the ileum of XCB piglets, as well as in the duodenum, ileum, and spleen of TYB piglets, but it up-regulated the *IGFBP-2* expression in the kidney of XCB and DR piglets and the LD muscle of DR piglets (*p* < 0.05). There were interactions (*p* < 0.05) between weaning and breed for *IGFBP-2* expression in the duodenum, ileum, kidney, spleen, liver, and LD muscle.

At 21 days of age, the *IGFBP-3* expression was up-regulated (*p* < 0.05) in the duodenum and liver of TYB piglets compared with the XCB and DR piglets. At 24 days of age, the *IGFBP-3* expression was down-regulated (*p* < 0.05) in the stomach of XCB and TYB piglets compared with the DR piglets. Weaning down-regulated the *IGFBP-3* expression in the duodenum and liver of TYB piglets, but it up-regulated the *IGFBP-3* expression in the stomach of DR piglets (*p* < 0.05). There were interactions (*p* < 0.05) between weaning and breed for *IGFBP-3* expression in the stomach, duodenum, and liver.

The *IGFBP-4* expression was down-regulated in the ileum and up-regulated in the liver of XCB and TYB piglets compared with the DR piglets at 21 days of age (*p* < 0.05). At 24 days of age, the *IGFBP-4* expression was down-regulated (*p* < 0.05) in the liver of XCB and TYB piglets compared with the DR piglets. Weaning down-regulated the *IGFBP-4* expression in the ileum of three breeds of piglets and the liver of XCB and TYB piglets, whereas it up-regulated (*p* < 0.05) the *IGFBP-4* expression in the duodenum and jejunum, regardless of breed, and the liver of DR piglets (*p* < 0.05). There were interactions (*p* < 0.05) between weaning and pig breed for *IGFBP-4* expression in the ileum and liver.

In comparison to the DR piglets, the *IGFBP-5* expression was down-regulated in the ileum but up-regulated in the kidney and liver of XCB and TYB piglets at 21 days of age (*p* < 0.05). The *IGFBP-5* expression was up-regulated (*p* < 0.05) in the duodenum and spleen of XCB piglets compared with the TYB and DR piglets, regardless of weaning. Weaning up-regulated the *IGFBP-5* expression in the ileum of DR piglets, as well as in the kidney and liver of XCB and TYB piglets, while it up-regulated the *IGFBP-5* expression in the stomach, spleen, and LD muscle of piglets, regardless of breed (*p* < 0.05). There were interactions (*p* < 0.05) between weaning and breed for *IGFBP-5* expression in the ileum, kidney, and liver.

At 21 days of age, the *IGFBP-6* expression was up-regulated (*p* < 0.05) in the liver of TYB piglets compared with the XCB and DR piglets, as well as in the LD muscle of XCB and TYB piglets compared with the DR piglets. At 24 days of age, the *IGFBP-6* expression was down-regulated (*p* < 0.05) in the stomach, jejunum, and ileum of XCB piglets compared with the DR piglets. Weaning down-regulated the *IGFBP-6* expression in the ileum and LD muscle of XCB and TYB piglets, while it up-regulated the *IGFBP-6* expression in the jejunum of TYB and DR piglets (*p* < 0.05). The *IGFBP-6* expression in the stomach, jejunum, ileum, liver, and LD muscle showed weaning–breed interaction (*p* < 0.05).

### 3.6. Weaning Effects on IGF Expressions in Different Tissues of the Same Breed of Piglets

**Figure 1:**
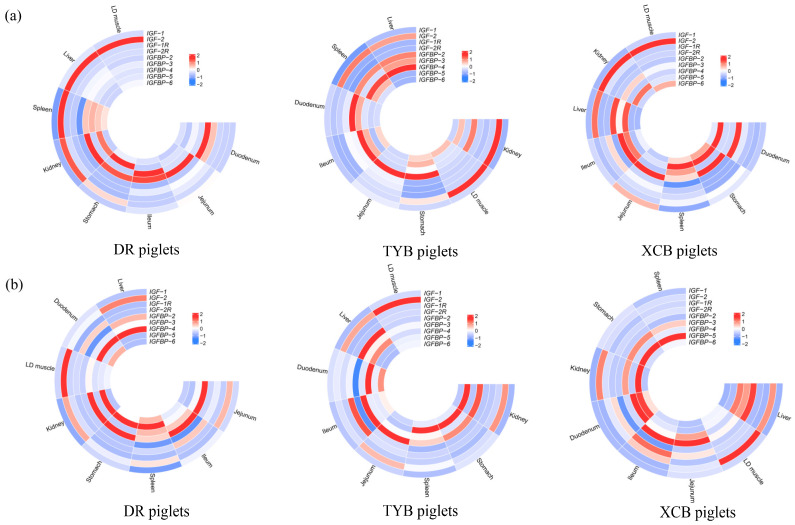
Expression of IGF-related genes in the *longissimus dorsi* (LD) muscle, liver, kidney, stomach, spleen, duodenum, jejunum, and ileum tissues of the same breed of piglets at 21 (**a**) and 24 (**b**) days of age. DR, Duroc piglet; TYB, Taoyuan black piglet; XCB, Xiangcun black piglet; *IGF*, insulin-like growth factor; *IGF-R*, insulin-like growth factor receptor; *IGFBP*, insulin-like growth factor binding protein. The red and blue in the figure represent the richness of gene expression, and the value is normalized.

The IGF expressions in different tissues of the same breed of piglets at 21 and 24 days of age are shown in Figure 1. At 21 days of age, the *IGF-1* expression in the liver of piglets was up-regulated (*p* < 0.05) in comparison to the other tissues. At 24 days of age, the *IGF-1* expression in the liver of DR piglets was up-regulated (*p* < 0.05) compared with the other tissues, as well as in the liver, stomach, and jejunum of TYB piglets and the stomach of XCB piglets.

At 21 days of age, the *IGF-2* expression was up-regulated (*p* < 0.05) in the liver and kidney of DR piglets, as well as in the liver of XCB and TYB piglets compared to the other tissues. At 24 days of age, the *IGF-2* expression was up-regulated (*p* < 0.05) in the liver of DR piglets and in the liver and kidney of XCB and TYB piglets compared to the other tissues.

The *IGF-1R* expression in the kidney of piglets was up-regulated (*p* < 0.05), as well as *IGF-2R* expression in the kidney of DR piglets compared with the other tissues at 21 and 24 days of age. At 21 days of age, the *IGF-2R* expression was up-regulated (*p* < 0.05) in the kidney and liver of TYB piglets, as well as in the liver of XCB piglets in comparison to the other tissues. At 24 days of age, the *IGF-2R* expression was up-regulated (*p* < 0.05) in the kidney of TYB and XCB piglets in comparison to the other tissues.

The *IGFBP-2* expression in the liver of piglets was up-regulated (*p* < 0.05), as well as *IGFBP-3* expression in the kidney of DR piglets compared with the other tissues at 21 and 24 days of age. At 21 days of age, the *IGFBP-3* expression was up-regulated (*p* < 0.05) in the liver of TYB and XCB piglets in comparison to the other tissues. At 24 days of age, the *IGFBP-3* expression was up-regulated (*p* < 0.05) in the kidney of TYB piglets, as well as in the liver and kidney of XCB piglets compared with the other tissues.

The *IGFBP-4* expression was up-regulated (*p* < 0.05) in the kidney of DR piglets at 21 days of age, as well as in the liver at 24 days of age. At 21 and 24 days of age, the *IGFBP-4* expression was up-regulated (*p* < 0.05) in the liver of TYB and XCB piglets, as well as *IGFBP-5* and *IGFBP-6* expressions in the kidney of piglets, when compared with the other tissues.

### 3.7. Correlations between Plasma Concentrations of IGF System Components, BW, and Organ Indices, as Well as IGF System Component Gene Expression and Organ Indices of Different Piglet Breeds

**Figure 2:**
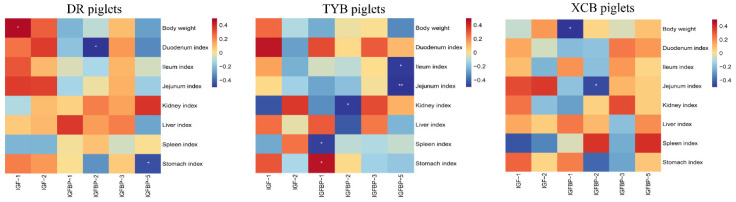
Spearman’s correlation analysis between body weight, organ indices, and plasma IGF system components in the three breeds of piglets. DR, Duroc piglet; TYB, Taoyuan black piglet; XCB, Xiangcun black piglet; IGF, insulin-like growth factor; IGFBP, insulin-like growth factor binding protein. The red and blue represent a significantly positive correlation and negative correlation, respectively. * *p* < 0.05, ** *p* < 0.01.

Spearman’s correlation analysis was performed to assess the correlations between plasma concentrations of IGF system components, BW, and organ indices of the three breeds of piglets (Figure 2). In DR piglets, the positive correlation included between plasma IGF-1 concentration and BW (*R* = 0.479, *p* < 0.05), and the negative correlations included between plasma IGFBP-2 concentration and the duodenum index (*R* = −0.530, *p* < 0.05) and plasma IGFBP-5 concentration and the stomach index (*R* = −0.461, *p* < 0.05). In TYB piglets, the positive correlation (*R* = −0.530, *p* < 0.05) included between plasma IGFBP-1 concentration and the stomach index (*R* = 0.444, *p* < 0.05), and the negative correlation included between IGFBP-1 concentration and the spleen index (*R* = −0.457, *p* < 0.05) of piglets. In addition, the negative correlations between plasma IGFBP-2 concentration and the kidney index (*R* = −0.481, *p* < 0.05) and plasma IGFBP-5 concentration and the jejunum (*R* = −0.654, *p* < 0.05) and ileum indices (*R* = −0.506, *p* < 0.05) were included in the TYB piglets. In XCB piglets, the negative correlations included between plasma IGFBP-1 concentration and BW (*R* = −0.553, *p* < 0.05) and plasma IGFBP-2 concentration and the jejunum index (*R* = −0.449, *p* < 0.05) of piglets.

**Figure 3:**
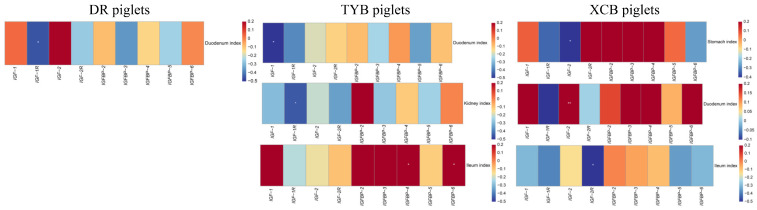
Spearman’s correlation analysis between organ indices and IGF system component gene expressions in the three breeds of piglets. DR, Duroc piglet; TYB, Taoyuan black piglet; XCB, Xiangcun black piglet; *IGF*, insulin-like growth factor; *IGFBP*, insulin-like growth factor binding protein. The red and blue represent a significantly positive correlation and negative correlation, respectively. * *p* < 0.05, ** *p* < 0.01.

The correlations between the IGF system component gene expressions and organ indices are shown in Figure 3. In DR piglets, the *IGF-1R* expression was negatively correlated with the duodenum index (*R* = −0.454, *p* < 0.05). In TYB piglets, the *IGF-1* expression was negatively correlated with the duodenum index (*R* = −0.513, *p* < 0.05), as well as the *IGF-1R* expression with the kidney index (*R* = −0.446, *p* < 0.05). *IGFBP-4* and *IGFBP-6* expressions were positively correlated with the ileum index (*R* = −0.494, −0.503, *p* < 0.05). In XCB piglets, the *IGF-2* expression was positively correlated with the duodenum index (*R* = 0.603, *p* < 0.05), whereas *IGF-2* expression was negatively correlated with the stomach index (*R* = −0.547, *p* < 0.05) and *IGF-2R* expression with the ileum index (*R* = −0.543, *p* < 0.05).

## 4. Discussion

Weaning is one of the most stressful events of the pig’s life, leading to a decrease in the growth rate [27]. The IGF system is essential for the growth and development of animals [28]. The effects of weaning on the IGF system in the TYB, XCB, and DR piglets were explored. We found that weaning affects the expressions of the IGF system components in piglets, and the gene expressions related to the IGF system differed among the three breeds of piglets.

The IGF system is fundamental for normal embryonic and postnatal growth. In addition, it plays an essential role in physiological functions such as the immune system, cell growth, myogenesis, and bone growth [29]. IGF-1 and IGF-2, two ligands of the IGF system, exert various functions in animals [30]. The IGF-1 circulates in the blood to regulate the overall growth rate, while IGF-1 within tissues enhances cellular growth, differentiation, and survival. Furthermore, IGF-1 influences glucose metabolism and has neuroprotective and cardioprotective effects [9]. The IGF-2 concentration during the prenatal period affects fetal growth and development. In addition, IGF-2 has important tissue-specific functions, such as maintaining the stem cell population and regulating muscle growth and differentiation [31,32]. In the present study, plasma IGF-1 concentration was higher in the XCB pigs than in the TYB and DR piglets. Our previous study showed that XCB and TYB piglets have lower BW while having higher nitrogen metabolism ability than the DR piglets [22]. A previous study reported that IGF-1 promotes protein synthesis [33]. Therefore, we speculated that the higher IGF-1 of XCB piglets is used for protein synthesis, while IGF-1 of DR pigs may be contributing to growth regulation. Further studies are needed to reveal the exact mechanism. In the present study, the effects of weaning on plasma IGF-1 and IGF-2 concentrations in different breeds of piglets were not significant. However, Matteri et al. [16] reported that weaning reduced serum IGF-1 and IGF-2 concentrations in pigs. These discrepancies might be explained by the pig breed, physiological stage, and experimental conditions.

IGF-1 and IGF-2 are essential for whole-body growth, physiology, and metabolism [31]. *IGF-1* and *IGF-2* expressions were mainly up-regulated in the liver of piglets in the present study, which is in agreement with a previous study [34]. Weaning decreased the *IGF-1* expression in the jejunum, spleen, and liver, as well as *IGF-2* expression in the kidney and spleen of piglets regardless of breed. These findings indicate that IGF expressions were reduced in organs with substance metabolism or immune function. Previously, it has been reported that weaning adversely affects the nutritional metabolism and immune function of piglets [35]. Moreover, Zhong et al. [36] found that IGF-1 injection promotes nutrient utilization and immune function in the stomach of aquatic animals. These results suggest that lower *IGF* expression may also affect digestion, absorption, and immunity in weaned piglets. The *IGF-1* expression was up-regulated in the stomach of XCB piglets, as well as in the stomach and jejunum of TYB piglets at 24 days of age in this study, suggesting that the XCB and TYB piglets may be more capable of resisting weaning stress than the DR piglets. Feng et al. [37] also reported that the proportion of the anti-stress sensitive gene genotype (Hal-NN) in Chinese indigenous pig breeds was significantly elevated compared with commercial pig breeds.

IGFs bind to IGFRs to activate the mammalian target of rapamycin or mitogen-activated protein kinase signaling pathways, leading to metabolic or mitogenic outcomes [38]. The IGF-1R exists in various cells and tissues and possesses a high binding affinity for IGF-1 and IGF-2 [39], while IGF-2R has no catalytic function and therefore does not activate intracellular signaling processes [40]. The combination of IGFs and IGF-1R is essential for regulating the growth, endocrine system, and metabolism of animals [41]. Reindl et al. [13] reported that IGFRs were expressed in the muscle, spleen, liver, kidney, intestine, and heart. In the present study, the *IGF-1R* expression was mainly up-regulated in the kidney of piglets, while it was up-regulated in the liver of XCB piglets compared with the DR piglets at 21 and 24 days of age. A previous study reported that the up-regulated *IGF-1R* expression markedly contributed to liver regeneration [42]. Those results are consistent with our previous findings, which indicated a higher liver index of the XCB piglets than the DR piglets at 21 days of age [26]. Overall, these findings support the hypothesis that up-regulated *IGF-1R* expression is one of the reasons for the strong anti-stress capability of Chinese indigenous pigs during weaning.

The activity of IGFs is mediated by the high affinity of IGFBPs that temporal-spatially maintain normal metabolism [43]. To prolong their half-lives and modulate tissue access, IGFBPs bind to IGF-1 and IGF-2, thereby regulating the IGF function. Six IGFBP isoforms (IGFBP-1−6) have distinct functions to activate or suppress the actions of IGFs [44]. IGFBP-3, one of the most abundant IGFBPs in the blood, binding approximately 90% of IGF-1 [45], can block IGF action and inhibit cell growth [46]. In the present study, plasma IGFBP-3 concentration in the TYB piglets was higher compared with the DR and XCB piglets at 21 days of age, which may be one of the reasons for the slow growth rate of TYB piglets. In addition, weaning reduced the plasma IGFBP-3 concentration in the TYB piglets, suggesting that TYB piglets can resist the adverse effects of weaning stress on growth.

IGFBPs predominantly inhibit IGF action and are expressed in several tissues. In the present study, *IGFBPs* expressions in different tissues differed with pig breeds, which may result in selection pressure on obtaining different phenotypes related to these pig breeds [11]. A previous study also reported that the changes in *IGFBPs* of calves were related to nutrient intake [47]. In the present study, weaning down-regulated *IGFBP-2*, *IGFBP-3*, *IGFBP-4*, and *IGFBP-6* expressions in specific tissues of the XCB and TYB piglets but up-regulated them in the DR piglets, suggesting that TYB and XCB piglets have higher anti-stress ability. Nishihara et al. [48] also indicated that down-regulated *IGFBP-2*, *IGFBP-3*, and *IGFBP-6* expressions could activate IGFs in rumen epithelial cells and promote papillae growth in weaned calves. It is worth noting that weaning up-regulated the *IGFBP-5* expression in several tissues of piglets in the present study. Previously, it has been reported that *IGFBP-5* expression was controlled by weaning in Japanese black calves [49]. However, *IGFBP-5* not only inhibits IGF action but also has the potential for IGF action [50]. Hence, the function of *IGFBP-5* needs to be further explored.

The IGF system regulates the growth and development of animals. In the present study, Spearman’s correlation analysis revealed that plasma IGF-1 concentration was positively correlated with the BW of the DR piglets, which was confirmed by the fact that DR pigs have a faster growth rate than Chinese indigenous pigs. In addition, plasma IGFBP-2 concentration was negatively correlated with the duodenum index of the DR and XCB piglets and the kidney index of the TYB piglets, as well as plasma IGFBP-5 concentration with the stomach index of the DR piglets and the jejunum and ileum indexes of the TYB piglets. These findings suggest that IGFBPs may regulate the growth and development of organs. In the present study, there was also a negative correlation between IGF-Rs and organ indices, which might be related to the compensatory response of piglets to weaning stress. However, further studies are necessary to explore the exact mechanism.

## 5. Conclusions

The tissue expression profiles of the IGF system components varied among different breeds of pigs, and IGFBPs have inhibitory effects on organ development. Weaning stress slowed down pigs’ growth by down-regulating the *IGF-1* expression. The expressions of IGF system components in response to weaning stress differed among breeds; particularly, *IGFBPs* expressions were down-regulated in the Xiangcun black and Taoyuan black piglets during weaning, which might be associated with the resistance response to the effects of weaning stress on slowing down the growth of piglets. Further studies are needed to confirm it. These findings have important implications for understanding the growth of different breeds of pigs during weaning and also provide a new perspective to alleviate the adverse effects of weaning stress on piglet growth.

## Figures and Tables

**Table 1:** Primer sequences used in this study.

Gene Names	Accession No.	Primer Sequences (5′-3′)	Product Size (bp)
*IGF-1*	XM_005664199.3	F: CCAAGGCTCAGAAGGAAGTACA	137
		R: ACTCGTGCAGAGCAAAGGAT	
*IGF-2*	NM_213883.2	F: CGGCTTCTACTTCAGCAGGC	219
		R: TGCTTCCAGGTGTCATAGCG	
*IGF-1R*	XM_021082915.1	F: ACGAGTGGAGAAATCTGCGG	154
		R: TGAGCTTGGGAAAGCGGTAG	
*IGF-2R*	NM_001244473.1	F: ACAGAAGCTGGACGTCATCG	150
		R: CTGTCAACGTCGAACCTGCT	
*IGFBP-1*	NM_001195105.1	F: CTATCACAGCAAACAGTGCGAG	181
		R: CACGTGAAGGAAGAGAGCCT	
*IGFBP-2*	NM_214003.1	F: CGAGCAGGTTGCAGACAATG	288
		R: GTGGAGATCCGTTCCAGGAC	
*IGFBP-3*	NM_001005156.1	F: AAGAAAAAGCAGTGCCGCC	208
		R: GATCGTGTCCTTGGCAGTCT	
*IGFBP-4*	NM_001123129.1	F: CTGCTCCGAAGAGAAGCTGG	279
		R: TCACCCTCGTCCTTGTCAGA	
*IGFBP-5*	NM_001315595.1	F: GCAAGCCAAGATCGAGAGAG	102
		R: GTGTGCTTGGGTCGGAAGAT	
*IGFBP-6*	NM_001100190.1	F: CCCTCGGGGGAGAATCCTAA	157
		R: GGCAAGGGCCCATCTCAG	
*β-Actin*	XM_0210860471	F: GATCTGGCACCACACCTTCTACAAC	107
		R: TCATCTTCTCACGGTTGGCTTTGG	

*IGF*, insulin-like growth factor; *IGF-R*, insulin-like growth factor receptor; *IGFBP*, insulin-like growth factor binding protein.

**Table 2:** Organ indices of the three breeds of piglets at 21 and 24 days of age.

Organ Indices, g/kg	21 Days of Age			24 Days of Age			SEM	*p*-Values		
	DR	TYB	XCB	DR	TYB	XCB		Weaning	Breed	W × B
Stomach	6.09	5.55	6.84	6.40	6.41	8.15	0.17	0.003	<0.001	0.322
Duodenum	0.56	0.61	0.83	0.56	0.62	0.66	0.03	0.262	0.013	0.270
Jejunum *	14.66	17.67	18.96	15.51	15.03	17.91	0.64	0.454	0.098	0.529
Ileum *	16.75	20.07	20.09	17.80	15.25	19.55	0.56	0.185	0.120	0.076
Kidney	6.04	6.21	6.21	6.01	5.82	5.94	0.09	0.230	0.961	0.729
Spleen *	2.08	2.04	2.18	1.93	1.87	1.75	0.07	0.074	0.948	0.646
Liver *	23.96	25.98	27.37	26.85	25.64	26.59	0.38	0.427	0.207	0.097

Data are expressed as mean ± SEM (*n* = 10). * Data obtained from previous studies [22,26]. DR: Duroc piglet; TYB: Taoyuan black piglet; XCB: Xiangcun black piglet.

**Table 3:** The plasma IGF and IGFBP concentration in different breeds of piglets at 21 and 24 days of age.

Index, µg/µL	21 Days of Age			24 Days of Age			SEM	*p*-Values		
	DR	TYB	XCB	DR	TYB	XCB		Weaning	Breed	W × B
IGF-1	15.95	15.17	17.88	15.50	15.06	17.86	0.39	0.797	0.012	0.971
IGF-2	1.67	1.61	1.79	1.79	1.53	1.83	0.05	0.799	0.152	0.740
IGFBP-1	38.90	42.82	43.29	44.42	45.41	42.31	1.42	0.425	0.794	0.668
IGFBP-2	17.82	22.30	28.76	18.40	22.57	22.12	0.99	0.277	0.006	0.177
IGFBP-3	36.53 ^b^	48.03 ^a^	35.66 ^b^	37.66 ^b^	34.05 ^b^	32.00 ^b^	1.35	0.029	0.065	0.045
IGFBP-5	12.46	17.27	23.14	11.52	17.91	21.81	0.82	0.588	<0.001	0.694

Data are expressed as mean ± SEM (*n* = 7–10). ^a,b^ Mean with different superscript letters within the same row indicates a significant difference (*p* < 0.05). DR: Duroc piglet; TYB: Taoyuan black piglet; XCB: Xiangcun black piglet; IGF: insulin-like growth factor; IGFBP: insulin-like growth factor binding protein.

**Table 4:** The gene expression of *IGFs* in tissues of the three breeds of piglets at 21 and 24 days of age.

Genes	Tissues	21 Days of Age			24 Days of Age			SEM	*p*-Values		
		DR	TYB	XCB	DR	TYB	XCB		Weaning	Breed	W × B
*IGF-1*	Stomach	1.03	2.10	2.13	2.29	1.66	1.88	0.19	0.610	0.735	0.134
	Duodenum	1.07	1.34	1.29	1.00	1.26	0.70	0.08	0.122	0.227	0.314
	Jejunum	1.60 ^abc^	1.86 ^ab^	2.52 ^a^	1.49 ^bc^	1.60 ^abc^	0.78 ^c^	0.15	0.011	0.841	0.030
	Ileum	0.78	0.61	0.68	0.78	0.34	0.33	0.04	0.003	0.001	0.101
	Kidney	0.90 ^a^	0.58 ^b^	0.43 ^b^	0.60 ^b^	0.89 ^a^	0.62 ^b^	0.04	0.308	0.015	0.002
	Spleen	0.26 ^b^	1.25 ^a^	0.84 ^a^	0.17 ^b^	0.34 ^b^	0.21 ^b^	0.08	<0.001	0.002	0.028
	Liver	5.80 ^ab^	7.66 ^a^	3.27 ^c^	3.77 ^bc^	1.75 ^c^	1.31 ^c^	0.45	<0.001	0.004	0.033
	LD muscle	0.28	0.34	0.33	0.41	0.43	0.33	0.03	0.169	0.729	0.630
*IGF-2*	Stomach	3.62	4.00	2.74	3.76	1.42	1.66	0.27	0.018	0.042	0.080
	Duodenum	1.07	0.97	1.12	1.13	1.35	0.67	0.09	0.985	0.440	0.158
	Jejunum	0.82	0.80	0.70	4.12	6.71	0.84	0.67	0.002	0.045	0.055
	Ileum	0.46	0.40	0.48	0.43	0.43	0.31	0.03	0.389	0.817	0.416
	Kidney	52.33	65.69	62.44	31.22	55.00	49.49	2.76	0.003	0.007	0.642
	Spleen	6.82	6.14	3.21	3.20	2.81	1.68	0.38	<0.001	0.001	0.269
	Liver	74.11	104.84	125.37	79.73	61.70	64.75	10.06	0.117	0.761	0.382
	LD muscle	12.82	11.71	11.81	16.91	16.86	9.35	0.99	0.244	0.152	0.225

Data are expressed as mean ± SEM (*n* = 6–8). ^a–c^ Mean with different superscript letters within the same row indicates a significant difference (*p* < 0.05). DR: Duroc piglet; TYB: Taoyuan black piglet; XCB: Xiangcun black piglet; *IGF*: insulin-like growth factor; LD: *longissimus dorsi*.

**Table 5:** The gene expression of *IGF* receptors in tissues of the three breeds of piglets at 21 and 24 days of age.

Genes	Tissues	21 Days of Age			24 Days of Age			SEM	*p*-Values		
		DR	TYB	XCB	DR	TYB	XCB		Weaning	Breed	W × B
*IGF-1R*	Stomach	0.49	0.65	0.61	0.57	0.31	0.51	0.04	0.179	0.787	0.136
	Duodenum	1.10	1.20	1.24	0.65	1.24	0.96	0.06	0.063	0.073	0.264
	Jejunum	1.18	0.99	0.92	1.16	1.22	0.51	0.08	0.671	0.052	0.271
	Ileum	0.45	0.40	0.62	0.50	0.37	0.63	0.04	0.927	0.023	0.866
	Kidney	11.63	8.27	10.64	11.74	12.06	13.71	0.45	0.006	0.116	0.154
	Spleen	1.35	1.77	2.41	1.05	1.08	1.27	0.13	0.003	0.070	0.312
	Liver	0.89 ^c^	2.28 ^a^	1.96 ^ab^	1.25 ^c^	1.80 ^b^	2.32 ^a^	0.10	0.521	<0.001	0.013
	LD muscle	0.22	0.09	0.25	0.36	0.26	0.23	0.03	0.147	0.372	0.467
*IGF-2R*	Stomach	0.63	0.75	0.68	1.18	0.96	1.41	0.07	<0.001	0.362	0.167
	Duodenum	1.06	1.81	1.65	1.41	1.42	1.23	0.09	0.387	0.245	0.159
	Jejunum	1.50	1.94	1.38	2.10	2.34	1.23	0.14	0.271	0.037	0.467
	Ileum	0.88	1.11	1.16	1.14	0.92	0.82	0.05	0.376	0.971	0.045
	Kidney	5.09 ^b^	4.82 ^b^	3.45 ^c^	6.75 ^a^	7.32 ^a^	8.05 ^a^	0.29	<0.001	0.781	0.007
	Spleen	1.43	1.47	1.93	1.72	1.29	1.89	0.08	0.877	0.027	0.437
	Liver	3.28	5.29	5.50	4.28	4.86	5.03	0.19	0.914	0.001	0.111
	LD muscle	0.39	0.16	0.54	0.72	0.51	0.38	0.06	0.168	0.350	0.167

Data are expressed as mean ± SEM (*n* = 6−8). ^a–c^ Mean with different superscript letters within the same row indicates a significant difference (*p* < 0.05). DR: Duroc piglet; TYB: Taoyuan black piglet; XCB: Xiangcun black piglet; *IGF-R*: insulin-like growth factor receptor; LD: *longissimus dorsi*.

**Table 6:** The gene expression of *IGFBPs* in tissues of the three breeds of piglets at 21 and 24 days of age.

Genes	Tissues	21 Days of Age			24 Days of Age			SEM	*p*-Values		
		DR	TYB	XCB	DR	TYB	XCB		Weaning	Breed	W × B
*IGFBP-1*	Liver	1.39	18.66	14.45	11.27	15.90	16.88	1.58	0.294	0.014	0.253
*IGFBP-2*	Stomach	0.66	0.93	0.54	1.03	0.65	0.92	0.06	0.207	0.753	0.056
	Duodenum	2.63 ^bc^	7.48 ^a^	3.38 ^b^	0.44 ^c^	0.51 ^c^	0.48 ^c^	0.53	<0.001	0.019	0.023
	Jejunum	0.48	2.23	0.70	1.39	2.06	0.58	0.20	0.568	0.004	0.406
	Ileum	0.16 ^b^	1.05 ^a^	0.33 ^b^	0.09 ^b^	0.15 ^b^	0.22 ^b^	0.08	0.016	0.031	0.039
	Kidney	0.34 ^c^	8.31 ^a^	5.44 ^b^	8.05 ^a^	6.60 ^ab^	8.24 ^a^	0.48	<0.001	<0.001	<0.001
	Spleen	0.05 ^b^	5.91 ^a^	0.12 ^b^	0.08 ^b^	0.09 ^b^	0.09 ^b^	0.35	<0.001	<0.001	<0.001
	Liver	10.36 ^c^	118.90 ^ab^	141.75 ^a^	60.67 ^bc^	111.30 ^ab^	85.11 ^ab^	9.88	0.775	<0.001	0.034
	LD muscle	0.02 ^b^	0.02 ^b^	0.03 ^b^	0.07 ^a^	0.05 ^ab^	0.03 ^b^	0.00	0.010	0.270	0.022
*IGFBP-3*	Stomach	6.50 ^b^	9.45 ^ab^	6.24 ^b^	14.92 ^a^	5.47 ^b^	6.72 ^b^	1.01	0.385	0.168	0.032
	Duodenum	1.14 ^b^	3.73 ^a^	1.47 ^b^	1.05 ^b^	1.30 ^b^	0.87 ^b^	0.22	0.004	0.002	0.018
	Jejunum	5.89	17.62	5.63	8.91	15.87	2.94	1.47	0.856	0.001	0.649
	Ileum	2.88	3.10	2.36	2.20	1.08	1.00	0.20	<0.001	0.146	0.314
	Kidney	58.21	42.01	25.75	47.70	69.12	44.26	3.62	0.075	0.023	0.056
	Spleen	3.78	2.98	4.30	3.48	4.33	5.42	0.29	0.212	0.151	0.440
	Liver	11.34 ^c^	105.14 ^a^	58.72 ^b^	33.50 ^bc^	37.02 ^bc^	62.67 ^b^	7.10	0.248	0.005	0.009
	LD muscle	1.11	0.33	2.09	3.28	2.27	1.86	0.33	0.047	0.485	0.241
*IGFBP-4*	Stomach	1.15	3.30	1.33	1.66	1.59	1.46	0.26	0.476	0.145	0.161
	Duodenum	1.28	1.66	1.48	2.47	3.01	1.87	0.19	0.008	0.306	0.505
	Jejunum	0.91	0.48	0.44	2.46	3.86	1.67	0.26	<0.001	0.095	0.082
	Ileum	3.68 ^a^	1.68 ^b^	1.68 ^b^	1.13 ^bc^	0.47 ^c^	0.53 ^c^	0.19	<0.001	<0.001	0.008
	Kidney	9.40	8.85	5.44	8.47	6.30	8.36	0.49	0.843	0.209	0.061
	Spleen	4.10	6.91	5.60	3.92	3.25	2.35	0.46	0.009	0.493	0.203
	Liver	6.05 ^c^	153.59 ^a^	136.24 ^a^	122.78 ^a^	70.81 ^b^	71.83 ^b^	9.25	0.405	0.005	<0.001
	LD muscle	0.66	0.77	0.53	1.10	0.62	0.34	0.08	0.833	0.074	0.179
*IGFBP-5*	Stomach	8.38	5.11	6.18	12.87	9.57	12.09	0.71	<0.001	0.097	0.856
	Duodenum	1.03	1.33	3.89	0.96	1.37	1.78	0.28	0.159	0.010	0.153
	Jejunum	1.01	0.85	0.95	0.86	0.96	0.53	0.06	0.156	0.314	0.161
	Ileum	0.48 ^b^	0.52 ^b^	0.49 ^b^	0.83 ^a^	0.46 ^b^	0.45 ^b^	0.04	0.224	0.064	0.030
	Kidney	47.43 ^b^	30.93 ^b^	42.19 ^b^	46.07 ^b^	81.66 ^a^	74.67 ^a^	3.66	<0.001	0.177	0.001
	Spleen	3.70	5.15	8.81	6.74	11.69	15.23	0.87	<0.001	0.001	0.508
	Liver	1.67 ^c^	1.28 ^c^	1.42 ^c^	2.01 ^c^	3.51 ^b^	5.75 ^a^	0.29	<0.001	0.001	<0.001
	LD muscle	0.90	0.88	1.22	1.59	1.83	1.35	0.13	0.020	0.937	0.377
*IGFBP-6*	Stomach	1.99 ^bc^	4.39 ^ab^	4.37 ^ab^	4.78 ^a^	1.84 ^c^	1.55 ^c^	0.38	0.220	0.880	0.002
	Duodenum	1.16	1.48	1.64	1.57	2.14	1.21	0.15	0.495	0.444	0.350
	Jejunum	0.64 ^b^	0.58 ^b^	0.55 ^b^	1.22 ^a^	1.70 ^a^	0.50 ^b^	0.10	0.001	0.007	0.014
	Ileum	0.74 ^b^	1.44 ^a^	0.85 ^b^	0.73 ^b^	0.57 ^bc^	0.41 ^c^	0.06	<0.001	0.001	<0.001
	Kidney	8.10	8.81	7.69	6.37	7.09	6.29	0.31	0.010	0.410	0.968
	Spleen	3.26	3.04	4.38	3.93	2.17	2.93	0.23	0.207	0.098	0.128
	Liver	1.52	1.13	1.08	1.14	1.59	1.62	0.08	0.177	0.990	0.033
	LD muscle	1.67 ^b^	3.99 ^a^	4.93 ^a^	2.21 ^b^	2.07 ^b^	1.27 ^b^	0.29	0.001	0.082	0.002

Data are expressed as mean ± SEM (*n* = 6−8). ^a–c^ Mean with different superscript letters within the same row indicates a significant difference (*p* < 0.05). DR: Duroc piglet; TYB: Taoyuan black piglet; XCB: Xiangcun black piglet; *IGFBP*: insulin-like growth factor binding protein; LD: *longissimus dorsi*.

## Data Availability

The data presented in this study are included in the article, and further inquiries can be directed to the corresponding author.
